# Screening programmes for developmental dysplasia of the hip in newborn infants

**DOI:** 10.1590/S1516-31802013000100028

**Published:** 2013-04-01

**Authors:** Damon Shorter, Timothy Hong, David A. Osborn

## Abstract

**BACKGROUND:**

Uncorrected developmental dysplasia of the hip (DDH) is associated with long term morbidity such as gait abnormalities, chronic pain and degenerative arthritis.

**OBJECTIVE:**

To determine the effect of different screening programmes for DDH on the incidence of late presentation of congenital hip dislocation.

**METHODS:**

*Search methods:* Searches were performed in CENTRAL (The Cochrane Library), MEDLINE and EMBASE (January 2011) supplemented by searches of clinical trial registries, conference proceedings, cross references and contacting expert informants.

*Selection criteria:* Randomized, quasi-randomized or cluster trials comparing the effectiveness of screening programmes for DDH.

*Data collection and analysis:* Three independent review authors assessed study eligibility and quality, and extracted data.

**MAIN RESULTS:**

No study examined the effect of screening (clinical and/or ultrasound) and early treatment versus not screening and later treatment.

**AUTHORS’ CONCLUSIONS:**

There is insufficient evidence to give clear recommendations for practice. There is inconsistent evidence that universal ultrasound results in a significant increase in treatment compared to the use of targeted ultrasound or clinical examination alone. Neither of the ultrasound strategies have been demonstrated to improve clinical outcomes including late diagnosed DDH and surgery. The studies are substantially underpowered to detect significant differences in the uncommon event of late detected DDH or surgery. For infants with unstable hips or mildly dysplastic hips, use of delayed ultrasound and targeted splinting reduces treatment without significantly increasing the rate of late diagnosed DDH or surgery.

**This is the abstract of a Cochrane Review published in the Cochrane Database of Systematic Reviews (CDSR) 2012, issue 11, Art. No. CD004595. DOI:** 10.1002/14651858.CD004595.pub2 (http://onlinelibrary.wiley.com/doi/10.1002/14651858.CD004595.pub2/abstract). For full citation and authors details see reference 1

**The full text is freely available, for Latin America and the Caribbean, from:** http://cochrane.bvsalud.org/cochrane/main.php?lib=COC&searchExp=Screening%20and%20programmes%20and%20for%20and%20developmental%20and%20dysplasia%20and%20of%20and%20the%20and%20hip%20and%20in%20and%20newborn%20and%20infants&lang=pt (this link may be temporary)
